# Stakeholder Perspectives on Cancer Survivors’ Return to Work and Well-Being: Qualitative Interview Study

**DOI:** 10.2196/89954

**Published:** 2026-05-11

**Authors:** Sara L Fällman, Katarina Wijk, Anna Efverman, Linda Eklund, Per Fessé, Sven Trygged, Maria Fjell

**Affiliations:** 1 Department of Caring Science, Faculty of Health and Occupational Studies, University of Gävle, Kungsbäcksvägen 47, Gävle, Gävleborg, 801 76, Sweden, 46 707305930; 2 Department of Work Life and Social Welfare, Faculty of Caring Science, Work Life and Social Welfare, University of Borås, Borås, Västra Götaland, Sweden; 3 Centre for Research and Development, Region Gävleborg, Uppsala University, Uppsala, Uppsala, Sweden; 4 Department of Occupational Health, Psychology and Sports Sciences, Faculty of Health and Occupational Studies, University of Gävle, Gävle, Gävleborg, Sweden; 5 Department of Public Health and Caring Sciences, Uppsala University, Uppsala, Sweden; 6 Center for Clinical Research Dalarna, Uppsala University, Uppsala, Sweden; 7 Department of Occupational Therapy and Healthcare Counselling, Region Dalarna, Ludvika, Sweden; 8 Department of Social Work, Criminology, and Public Health Science, Faculty of Health and Occupational Studies, University of Gävle, Gävle, Gävleborg, Sweden; 9 Division of Nursing, Department of Neurobiology, Care Sciences and Society, Karolinska Institutet, Stockholm, Sweden

**Keywords:** cancer survivors, rehabilitation, return to work, sickness absence, work participation

## Abstract

**Background:**

About 4 in 10 people in Sweden get cancer during their lifetime, and approximately half of them will be diagnosed during their working life. As cancer survival rates improve, a growing number of individuals face challenges in returning to work following treatment. This increases the demand for effective return-to-work (RTW) strategies. Despite existing rehabilitation frameworks, cancer survivors often encounter barriers to sustainable work reintegration.

**Objective:**

This study aimed to investigate stakeholders’ perceptions of facilitators and barriers to RTW among cancer survivors, including factors promoting work-related well-being during the RTW process.

**Methods:**

During the development phase of a work-oriented rehabilitation intervention, semistructured interviews were conducted, with 25 stakeholders involved in the RTW process: health care professionals (n=12), social insurance officers (n=7), employers (n=5), and an employment service agency officer (n=1). Data were analyzed using qualitative content analysis.

**Results:**

Five overarching themes were identified that influenced RTW: collaboration and clear division of responsibilities, balancing individual adaptations, reducing structural barriers through support, views and expectations of the individual regarding RTW, and the emotional significance of work. Key facilitators included flexible work arrangements, individualized adaptations, a strong desire to work, and the emotional value of work. Barriers comprised lack of collaboration among stakeholders, particularly the absence of a clear division of responsibilities, as well as structural barriers, such as sick leave bureaucracy and financial obstacles. The role of rehabilitation coordinators was highlighted as pivotal in bridging gaps between stakeholders and ensuring continuity in care.

**Conclusions:**

RTW for cancer survivors is a complex, multifactorial process requiring coordinated efforts across health care, insurance, and employment sectors. Enhancing collaboration, clarifying stakeholder responsibilities, and implementing flexible, individualized support structures are essential to facilitating cancer survivors’ RTW. Additionally, including a designated coordinator in the process is proposed. More support during the early phase of RTW is necessary to reduce the risk of long-term labor market exclusion. To help cancer survivors’ RTW, clear role definitions and shared responsibilities among stakeholders are essential. Flexibility in the RTW process helps individuals reintegrate at their own pace, reduces isolation, and promotes social connection. These are key considerations for future policies and support measures.

## Introduction

When developing work-oriented rehabilitation interventions, it is crucial to consider the experiences of stakeholders in their working life. Understanding barriers and facilitators can enhance successful implementation of such interventions into practice [1,2]. Over the past 30 years, the global incidence of cancer among individuals younger than 50 years has risen by 79%. This trend is particularly pronounced in high-income countries, influenced by lifestyle factors and enhanced diagnostic methods [3,4]. Concurrently, advancement in cancer treatment has markedly improved survival rates for many cancer types, especially those affecting working-age populations [5]. In Sweden, it is noteworthy that almost half of all cancer cases are diagnosed during people’s working years [6]. Consequently, cancer survivors, individuals diagnosed with cancer who are still alive, are likely to remain active on the labor market for many years to come [7]. This reality underscores the importance of facilitating return to work (RTW) postdiagnosis [8-11]. Furthermore, cancer diagnoses have significant economic consequences for both individuals and society. Patients frequently experience considerable financial strain due to lost income from prolonged sick leave or reduced working hours [9]. On a societal level, the economic impact of cancer-related work absence in Sweden is substantial, estimated at approximately US $4 million annually, which is comparable with the cost associated with outpatient cancer care [12].

Although cancer treatment has improved survival, many survivors experience long-term physical, cognitive, and psychological effects that can hinder individuals’ work ability. Although more cancer survivors return to work [13], many face functional limitations [14,15], and about 25% of cancer survivors did not return even 14 years postdiagnosis [16]. Among those who do not return, many work part-time [17] or attribute their absence to mental illness, chronic pain, or musculoskeletal disorders [18]. In the Nordic countries, 14% of cancer survivors received disability pension 7 years after diagnosis [7], and many reported reduced work ability (K Tödt et al, unpublished data, 2026). Previous studies of common cancer types in Sweden, including breast, colorectal, and prostate cancer, have demonstrated that many survivors experience prolonged work absence following diagnosis and treatment. More recent evidence from a nationwide registered-based prospective cohort study showed that, even after returning to work, cancer survivors had a substantially higher risk of future labor market marginalization [19]. Specifically, they exhibited a 1.6-fold increased risk of new sick leave episodes, a 2-fold higher risk of receiving a disability pension, and a 1.3-fold increased risk of unemployment compared with matched individuals with no cancer [19]. These findings underscore the need for a comprehensive rehabilitation approach, viewing RTW as a complex process requiring coordinated medical, psychosocial, and work-oriented rehabilitation [20].

Prior research has shown that many cancer survivors are motivated to maintain or resume employment during and after treatment [13,21]. Prolonged absence from work is associated with reduced quality of life [22] and may erode dignity, social identity, and financial security [23,24]. These challenges have intensified calls for more effective multiple-stakeholder interventions to support occupational reintegration [8-11].

Motivation to return to work is shaped by both personal and contextual factors. Social support from family, peers, colleagues, and employers reinforces a sense of belonging and security, thereby facilitating labor market reintegration [8,11]. Equally critical are workplace conditions: flexible work arrangements, task modifications, and organizational cultures oriented toward inclusivity and health promotion, which are consistently identified as facilitators of RTW [7,9]. Collectively, the evidence underscores the importance of supportive networks and enabling organizational structures to reclaim their professional role following treatment.

Theories concerning cancer and work emphasize the importance of tailoring rehabilitation interventions to the individual cognitive and emotional resources of survivors, as well as to the specific demands they face related to their illness, treatment, work, and personal circumstances [20]. In non–cancer populations with, for example, musculoskeletal or mental illness disorders, the most promising interventions for RTW were found to be multidisciplinary, involving several stakeholders [11,25]. Reviews have shown positive effects of cancer rehabilitation in general, but several areas remain underdeveloped, particularly regarding RTW [15,26]. Cancer rehabilitation seeks to prevent and alleviate cancer-related consequences [15,26], while work-oriented rehabilitation specifically addresses those affecting work participation [10].

Approaches to cancer rehabilitation and RTW vary considerably across European countries. Differences in health care and social insurance systems, as well as in the distribution of responsibilities among stakeholders, shape both the design of interventions and how they are perceived by employers, health care providers, and insurance agencies [16,27,28]. Research highlights the importance of adapting rehabilitation strategies to each sociopolitical and institutional context, while also addressing the specific needs of cancer survivors and involved stakeholders [8,9,11]. The Swedish context illustrates this well. Employers are liable for wage compensation during the first initial 14 days of sick leave, after which financial benefits are administered by the Swedish Social Insurance Agency. Employers are responsible for workplace-related interventions, whereas the Swedish Social Insurance Agency coordinates actions among relevant authorities and stakeholders.

Important stakeholders regarding work participation include health care professionals in cancer, primary and occupational health care, managers, and social insurance officers [29-31]. Previous research has stressed that cancer survivors often require more comprehensive support from these diverse working-life stakeholders to facilitate a sustainable RTW process [21].

To enhance intersectoral collaboration, the Act on Coordination of Rehabilitation Efforts (Act no. 2019:1297) mandates the establishment of rehabilitation coordination. These professionals, commonly occupational therapists, physiotherapists, social workers, or registered nurses, serve as intermediaries between patients and key actors within the RTW process [32]. Their responsibilities include providing individualized support, ensuring communication across organizational boundaries, and facilitating the development of personalized rehabilitation plans [33]. At the core of the Swedish RTW system lie systematic assessments of work ability and structured measures aimed at promoting workplace reintegration. Collectively, these arrangements are intended to provide a coordinated and equitable approach to supporting individuals on sick leave.

Although the Act on Coordination of Rehabilitation Efforts requires regions to provide coordinated rehabilitation, these are often delivered through rehabilitation coordinators whose roles and visibility vary across settings [32,33]. Research suggests that coordinators may support RTW processes through continuity and coordination [20], but evidence of their effectiveness in improving RTW outcomes is inconsistent [10]. Although rehabilitation coordinators represent one important stakeholder in RTW processes, this study adopts a broader perspective to capture how facilitators and barriers emerge across multiple interacting actors.

Previous research has identified various facilitators and barriers to RTW after cancer [9,34,35], but much of this evidence is based on heterogeneous contexts [9] and overlooks how institutional conditions shape the RTW processes [16,36]. This is particularly relevant in Sweden, where RTW involves multiple actors across health care, workplaces, and the social insurance systems [17,19]. Key stakeholders’ perspectives also remain underexplored [23,31,37], especially regarding how these actors interact in practice [38], an important gap given that RTW is a multiactor process. Recent structural changes, such as the introduction of rehabilitation coordinators, have further reshaped RTW processes [32,33], yet limited empirical knowledge exists about how stakeholders experience these changes [21,39]. Against this backdrop, this study was conducted to generate context-specific, empirical insights into facilitators and barriers to RTW after cancer from a multistakeholder perspective. The study contributes by (1) integrating multiple stakeholder perspectives, and (2) informing the development of a tailored intervention.

Hence, it is essential to further explore and describe the perspectives of stakeholders themselves, as their roles, perceptions, and interactions are central to shaping the conditions under which cancer survivors attempt to resume working life. The aim of this study was to investigate working-life stakeholders’ perceptions of both facilitators and barriers to RTW among cancer survivors, including factors for work-related well-being during the RTW process.

## Methods

### Study Design

This qualitative study was a part of the development phase of a complex intervention following the updated Medical Research Council framework [40] regarding work-oriented rehabilitation. A qualitative interview design was chosen to gain an in-depth understanding of stakeholders’ experiences and perceptions of cancer survivors’ RTW process. Semistructured interviews were conducted with 25 stakeholders representing various roles in the RTW context during spring 2023. The qualitative interview approach provided flexibility and allowed participants to elaborate on issues of personal and professional relevance [41].

Sample participants were recruited using purposive sampling to ensure variation in characteristics, geographic locations, and work settings (specialized care, primary care, and public and private sector). Purposive sampling involves the intentional selection of information-rich cases to capture diverse experiences related to the studied phenomenon [42]. The sampling aimed to capture variation both between stakeholder groups and within groups comprising multiple roles. For example, the health care professional group included several relevant clinical disciplines (physicians, nurses, occupational therapists, and physiotherapists). The sampling strategy also reflected the stakeholders commonly involved in RTW processes for cancer survivors, resulting in higher representation of health care professionals and representatives from the Swedish Social Insurance Agency, while representation from Employment Agency officers was more limited (n=1). Rehabilitation coordinators were included as one of several stakeholder groups; however, the study was not designed to specifically examine their role but rather to explore facilitators and barriers to RTW across multiple stakeholders.

Participants represented 3 regions in central Sweden, involved in a larger research program developing an intervention to support cancer survivors’ RTW. Stakeholders included health care practitioners (registered nurse, occupational therapist, physician, counselor, and rehabilitation coordinator), social insurance officers, employment service agency representatives, and first-line managers from private and public employers. Selection was based on experience supporting cancer survivors in maintaining or returning to work during cancer treatment. Inclusion criteria were employment within a stakeholder group relevant to RTW processes (ie, health care professionals, social insurance officers, employment service officers, social workers, or first-line managers) and experience of supporting cancer survivors in RTW. No specific exclusion criteria were applied; however, all participants were required to be able to participate in an interview conducted in Swedish. The study followed COREQ (Consolidated Criteria for Reporting Qualitative Research) [43].

### Setting

The study was conducted within the Swedish RTW system, a tax-funded framework involving health care services, employers, and the Swedish Social Insurance Agency. Employers provide sick pay during the initial 2 weeks of sick leave, after which the Swedish Social Insurance Agency assesses work capacity, administers benefits, and coordinates rehabilitation in collaboration with health care providers. Employers are also responsible for workplace-based rehabilitation, while additional support, such as rehabilitation, may be provided by the Swedish Public Employment Service (Act no. 2019:1297).

RTW processes typically involve ongoing interaction between these stakeholders, where health care providers assess work ability, employers implement workplace adjustments, and the Social Insurance Agency coordinates measures and monitors eligibility for benefits. For individuals with cancer, the system includes specific regulations and coordinates efforts intended to support a gradual and individualized RTW process, although the level of coordination may vary.

### Data Collection

A study coordinator recruited participants in 2 stages. First, brief study information was emailed to key organizational contacts relevant to the study, who were asked to forward the invitation to potential participants. Interested individuals contacted author SLF for further information. Subsequently, comprehensive information was provided, eligibility criteria were verified, and participants were informed about the purpose of the study. Initially, 26 individuals expressed interest in participating; however, 1 participant withdrew prior to the interview due to personal reasons, resulting in 25 completed interviews. Participants provided informed consent and, immediately before the interview, completed a brief web-based survey describing their demographic characteristics (Table 1). Participants’ age ranged from 39 to 65 years, and those who were employers had between 8 and 46 employees (median 43).

**Table 1.** The participating working-life stakeholders.

Variable	Participants (n=25)
Age (years), median (25th to 75th percentile)	48 (43‐59)
Gender, n (%)	
Men	4 (16)
Women	21 (84)
Occupational sector, n (%)	
Health care^a^	12 (48)
Social Insurance Agency	7 (28)
Employment Service Agency	1 (4)
Employer^b^	5 (20)
Years of occupational experience^c^, median (25th-75th percentile)	18 (5‐25)
Frequency of working with people with cancer, n (%)	
Several times a day	7 (28)
Once a day	2 (8)
Once a week	4 (16)
Once a month	4 (16)
Several times a year	4^d^ (16)
Less often than once a year	4^e^ (16)
Knowledge about the national guideline recommendations on cancer rehabilitation^f^, n (%)	
No, I don’t know about it	6 (24)
Yes, but I have not read it	14 (56)
Yes, I have read it	5 (20)

a Their occupations were nurse (n=3), occupational therapist (n=3), physician (n=3), counselor (n=1), and rehabilitation coordinator (n=2). The health care settings were specialized care (n=9) (eg, oncology: n=4), primary care (n=2), and occupational health care (n=1).

b The employers worked in the public sector (n=3), private sector in a middle-sized company (n=1), or private sector in a big company (n=1).

c In their present occupation.

d Being health care professionals within primary care or rehabilitation clinics.

e Being employers.

f Distributed by the Confederation of Regional Cancer Centres [44].

The authors SLF, LE, PF, and MF interviewed the stakeholders individually, one-on-one in a video meeting. The interviewers comprised a district nurse, an occupational therapist, and 2 oncology nurses; 3 were women and 1 was a man. The interviewers had both professional experience in clinical cancer care and experience in conducting qualitative interviews. A semistructured interview guide was followed (Textbox 1), asking follow-up questions as needed to deepen or expand responses, in line with qualitative interview methodology [41]. Interviews were audio-recorded, lasting between 20 and 71 minutes (median 42 minutes).

Textbox 1. **The semistructured interview guide.**

At the beginning of the interview, the interviewer introduced themselves and briefly described their professional background. The interviewer presented a case study based on a fictional patient “Kim” on whom the informants were asked to base their reasoning. The first questions were connected to this case, and the case description formed the basis for the interview. In the last questions of the interview, we moved away from the Kim case and asked interviewees to talk more generally about the questions. When necessary, field notes were made. A detailed description of the case used during the interviews is provided in Multimedia Appendix 1.

Interview questions

- What challenges do you anticipate regarding work in relation to having cancer?
- Could you tell me if you have a role in supporting individuals’ work and occupational health during or after cancer treatment, and if so, what does this involve?
- Can you tell me which other actors have roles here?
- What do you believe could promote work and health here, and what do you believe could hinder work and health?
- Does Kim, in this example, need any support regarding work and health? If so, please describe it.
  Moving beyond the case of Kim
- If anything were possible, what kind of support would need to exist to support people’s work and health during and after treatment?
- As a final question, we have now discussed...(your role, other stakeholders, collaboration, and support related to work and health). Is there anything else you would like to add regarding work or health in relation to work for individuals during or after cancer treatment?

### Data Analysis

According to Elo and Kyngäs [45], the interviews were transcribed verbatim and analyzed using qualitative content analysis. Initially, transcripts were read several times by authors SLF, KW, and MF to gain familiarity. No dedicated qualitative analysis software was used. Instead, data were managed in Microsoft Excel to organize transcripts, track codes, and collate categories. Data handling followed strict standards of integrity and confidentiality.

Transcripts were evenly distributed among these authors, who independently extracted meaning units relevant to the study aim and generated initial categories and subcategories. Subsequently, these authors collaboratively reviewed their analyses, refined categories and subcategories, and reached consensus on findings. All 25 interviews were analyzed as a single dataset. No separate analyses were conducted by stakeholder groups; instead, themes were developed based on patterns identified across the entire material. During coding, sector-related nuances were noted and considered in the interpretation, but no formal comparison between stakeholder groups was performed. An example of the analytic structure is illustrated by the data unit “Most people want to work. I have to say it’s an extremely exciting experience working with people, that everyone is so eager to get back to work. I think so.” which was coded as “Most people want to work,” grouped into the subcategory “Desire to work,” and further abstracted into the category “Emotional significance of work.” Trustworthiness was ensured through analyst triangulation to enhance credibility and reduce interpretative bias [42]. Dependability and confirmability were supported through transparent documentation and regular discussion within the research team [46]. Reflexivity and methodological transparency were emphasized to strengthen credibility and transferability, in line with qualitative research standards [41]. The research team included members with backgrounds in nursing, health-related research, and occupational therapy, which contributed to multiple professional perspectives during data interpretation. Reflexive discussions throughout the analytic process increased awareness of potential preunderstandings and supported interpretative rigor.

### Ethical Considerations

The study was approved by the Swedish Ethical Review Authority (registration numbers 2022-03686-01, 2023-05671-02) and conducted in compliance with the Declaration of Helsinki (World Medical Association, 2013). Informed consent was obtained from all participants. Participants did not receive any financial or material compensation for their participation.

## Results

### Overview

Barriers and facilitating factors regarding RTW were identified not only in the individual adaptations made by cancer survivors but also in cooperation among stakeholders. Structural issues, including financial obstacles and sick leave regulations, also influenced RTW outcomes. Highlighted factors promoting work-related well-being were flexibility and personalized adaptations. Categories and subcategories are presented in Table 2.

**Table 2.** Categories and subcategories of stakeholders’ perceptions of both facilitators and barriers to work among cancer survivors in a return to work process.

Category	Subcategory
Collaboration and clear division of responsibilities	Desire for developed and improved cooperation Who is responsible for what? Clear division of responsibilities
Balancing individual adaptations	Adaptations to the work situation Flexibility in RTW^a^ A balancing act
Reducing structural barriers through support	Financial obstacles to RTW Challenging sick leave bureaucracy
Views and expectations of the individual effect of RTW	Courage to push and set demands Inclusion in the workplace
Emotional significance of work	Desire to work Existential questions take focus

a RTW: return to work.

### Collaboration and Clear Division of Responsibilities

The interviews revealed not only a strong desire for more developed and improved collaboration among stakeholders but also a lack of awareness about available support and confusion over roles and responsibilities. Stakeholders emphasized the need for a structured collaboration plan to clarify roles and enhance teamwork. This, according to them, would facilitate collaboration and ensure that each party had a clear understanding of their respective roles and responsibilities.

#### Desire for Developed and Improved Cooperation

The informants stressed that cooperation between stakeholders sometimes had been lacking or functioned poorly, negatively affecting the RTW process. They described an impermeable silo mentality among stakeholders who were expected to collaborate, causing irritation and frustration over this lack of coordination, emphasizing that it had hindered efficiency in helping individuals return to work.

*I often get nasty answers and a feeling they think I’m not knowledgeable and that I disrupt contact with healthcare. That can be a barrier to returning to work*.

[12, Social Insurance Agency]

Throughout the interviews, a desire to increase and improve cooperation emerged. Informants strongly believed that closer collaboration is crucial for addressing the challenges and would lead to better, more coordinated outcomes. They noted that greater cooperation would have benefited cancer survivors’ RTW and resulted in better, faster, and more cost-effective care. Furthermore, informants also argued for a unified body to oversee the overall coordination.

They felt that moving away from siloed work structures toward team-based collaboration would be better for everyone and more cost-effective for society. Teamwork was seen as promoting continuity and reducing vulnerability, since remaining members could ensure stability even if 1 key person was replaced.

*We need more cooperation in teams. The healthcare system would save a lot of money if we got out of our silos, if we worked in teams, which I’ve had the benefit of doing at times. Because then you really can help people, compared to getting help from a physical therapist here, then a doctor and then a nurse, an occupational therapist, a psychologist. And then they don’t talk to each other*.

[6, occupational therapist]

Informants working in inpatient hospital care described well-functioning collaborations and established team models, but in other areas there was a total lack of collaboration between specialties. They emphasized the importance of enhanced collaboration, suggesting specialized outpatient teams with in-depth knowledge of diseases, as this might be difficult for primary care to manage on their own. They also recommended appointing a dedicated coordinator to oversee and support the entire RTW process and stressed the need for clear consensus and direction from higher leadership levels. Rehabilitation coordinators were highlighted as important facilitators of collaboration and serve as a link supporting cancer survivors’ RTW.

#### Who Is Responsible for What?

Among the informants, there was a perceived lack of knowledge about who was responsible for what, which they believed hindered the RTW process. More broadly, they noted an insufficient understanding of the tasks and roles of different professional groups and their potential contributions. This lack of clarity and knowledge was seen as a cause of failed initiatives, thereby negatively affecting the process. Furthermore, interviews revealed a lack of trust in the competence of certain individuals expected to participate in the sick leave process.

*...something I wonder about is that the person who’s supposed to decide on the sickness benefit isn’t required to have any knowledge at all of healthcare but is responsible for coordination*.

[19, manager]

Informants emphasized frequent mistreatment by the Social Insurance Agency. Some described such negative experiences that they questioned the agency’s role and purpose in the sick leave process. They believed that this situation imposed further barriers to the RTW process for cancer survivors.

*The collaboration between actors is dysfunctional—the Social Insurance Agency ignores us when we contact them; I’m not sure what they’re good for*.

[10, physician]

On the other hand, it was mentioned on several occasions that Social Insurance Agency employees felt that they were treated poorly, particularly by doctors.

*Well, it’s just that you feel you don’t know anything and that you’re a bother. Unfortunately, with time I’ve gotten quite a few sarcastic answers when I’ve...or supplemented a doctor’s certificate that I should have understood*.

[12, Social Insurance Agency]

They described experiencing a sense of being a disturbance and sometimes having been given substandard sick leave documentation to manage. When they asked supplementary questions, they felt that their inquiries were considered foolish and that they were expected to know more.

#### Clear Division of Responsibilities

During the interviews, the informants reported a lack of division of responsibilities and expressed a desire for greater clarity around who was responsible for what. They emphasized that lack of clarity regarding division of responsibilities prolonged the RTW process or, in some cases, prevented it altogether.

*I said she should do work training, but her employer was on vacation, so I had to put her on full-time sick leave // --- // I had to change her sick leave several times. If the Social Insurance Agency had contacted me instead, it would have been much simpler than having a patient try to explain*.

[8, physician]

There was a desire for a holistic approach to RTW that also considered social aspects. Doctors reported having control over medical issues but noted that the social perspective received far less attention due to time constraints. They believed that the process could have been facilitated and improved if a more holistic approach had been applied. Informants repeatedly stated that a specialized unit focusing on RTW for this group would have facilitated the process. The need for such a unit was further emphasized by those who described the situation as complex. They explained that doctors were sometimes expected to issue sickness certificates even when they were no longer involved in the patient’s treatment and had limited knowledge of, or contact with, the patient.

*Then there’s another aspect I think is sometimes a bit complicated. It’s that a lot of follow-ups occur through me, where they don’t have a doctor to contact, but anyway it’s the doctor who’s supposed to be behind the sick leave, which can sometimes cause confusion*.

[2, occupational therapist]

To improve efficiency and clarify responsibilities, informants suggested involving certain functions earlier in the process and assigning them a more defined role as a consistent link throughout the process. They emphasized the need for a dedicated person to follow up with the patient beyond the acute stage, indicating that the rehabilitation coordinator was well suited for this role.

### Balancing Individual Adaptations

The stakeholders reported that a flexible RTW approach was required to facilitate cancer survivors’ RTW process during and after cancer treatment, where tasks were adapted to cancer survivors’ abilities and needs. They said the process can be compared with a balancing act that requires continual adjustment in how it is set up.

#### Adaptations to the Work Situation

To facilitate a smooth and sustainable RTW process, informants reported tailoring the work situation to individuals’ needs. They emphasized adapting tasks and establishing a plan that ensures long-term sustainability for cancer survivors. Gradually increasing workload, based on a plan developed with the cancer survivor, was seen as essential. They also stated that adaptations may be necessary if cancer survivors are unable to handle excessive sensory input or work in large groups.

Informants noted that it is the manager’s responsibility to modify the work situation and ensure a workable solution. Specific adaptations could include adjusted working hours or a condensed schedule for additional recovery time. Informants indicated that, although most employers recognized the need and were willing to make individual adjustments, ongoing challenges persisted. The time required to identify alternative solutions for employees varied, and in recent years the process has sometimes been more challenging and restrictive. Some informants perceived a reluctance among employers to adapt to cancer survivors’ working situation.

*Maybe returning to work full-time isn’t doable, so instead you get to see if there are alternative solutions. But you’re always thinking along with colleagues and maybe others, so that you always have a plan, continuous conversations, follow-ups, so that all parties are agreed on the direction we’re going in*.

[21, manager]

There were different opinions about how flexible the system is in helping cancer survivors return to work after treatment. Some felt that making individual adjustments had become easier, while others thought that it had become more difficult due to changes in legislation. The bottom line, however, is that everyone felt that being able to make flexible adjustments was important.

#### Flexibility in RTW

Although work tasks were adapted to cancer survivors’ needs, informants noted that plans often required rebalancing as the survivors’ requirements changed after returning to work. Flexible tasks include the possibility to take short breaks during the day, while adjustment and shielding were sometimes necessary for those with treatment-related immune weakness, increasing vulnerability to infection. Consequently, cancer survivors might need to influence aspects of their work situation to ensure their own health and safety.

*Well, sometimes people’s immune system is suppressed due to treatment, or lack of treatment. So, then we’ve...Well, not discouraged her, but said you can do that from home during this period, that would be good, to avoid possible risks, like*.

[21, manager]

Employees may also have the flexibility to be at the workplace at times that suit them, with the option to work remotely and participate in meetings virtually.

#### A Balancing Act

Regarding the RTW process, the informants emphasized that it is a balancing act that affects how successful RTW and the process will be. They noted that planning is easy, but that checking progress along the way is often neglected. That neglect often undermines a sustainable RTW. Without regular check-ins, the likelihood of a successful transition decreases. It is crucial to navigate the process by finding the right balance between pushing forward and pulling back when needed.

*In my experience, it’s easy to push things and go too fast*.

[19, manager]

The informants found it important to be aware of physically and mentally demanding tasks and to monitor cancer survivors’ progress, checking how they manage these responsibilities. Furthermore, they emphasized the need to avoid putting excessive pressure on cancer survivors, while still valuing and using their skills and expertise. Finding this balance is essential and plays a significant role in ensuring a sustainable RTW.

### Reducing Structural Barriers Through Support

One area the interviewees felt had worsened concerned the structural barriers that could arise in connection with RTW. Among other things, they thought that regulation of working hours for RTW was a problem. They also pointed to the financial situation that could arise when employees wanted to return to work but for various reasons were unable to perform their regular tasks, as this could lead to increased costs. Furthermore, they stressed that lack of qualified access was an obstacle to creating a “smooth” RTW process.

#### Financial Obstacles to Returning to Work

Informants reported that patients’ financial situation could be a barrier to RTW. A key challenge was that employees were often unable to perform their pre-illness tasks. Even when employees could not carry out their duties fully, employers were still required to cover the costs, creating difficult circumstances. This could result in a situation where patients wanted to return to work, but employers could not afford to accommodate them. Conflicts sometimes arose when employees believed that they could work while employers doubted their ability.

Refusals or dismissals in such cases were described as more common among larger employers who, despite greater resources, still struggle to make necessary adjustments. Similarly, physically demanding occupations, such as industrial work, often faced considerable challenges in providing suitable accommodation. Nonetheless, many employers, despite lacking financial support, allowed employees to return even when not fully able to work, demonstrating notable commitment and fostering a more inclusive work environment.

*I think it’s pretty great that so many employers take them back, because they don’t gain anything; they’re just helping out. People need different degrees of support, but just having a supervisor, a specific person, I think that’s a good foundation for things working out*.

[18, Employment Service Agency]

One proposal that emerged in the interviews was that employers who accepted employees who were not yet fully able to work should receive financial support. The informants emphasized the importance of individuals feeling needed and having a role, without feeling like a financial burden on the employer.

#### Challenging the Sick Leave Bureaucracy

There was a broad consensus among informants that simplicity and flexibility in sick leave regulations were health-promoting, although not all agree. While many acknowledged recent simplification efforts by the Social Insurance Agency, others remained critical. They also highlighted the difficulties cancer survivors face when dealing with RTW regulations. One example concerned the requirement to distribute reduced working hours evenly across the week, which they viewed as impractical for cancer survivors able to work only brief periods; for example, 25% of working time equated to less than 2 hours a day. The informants described the regulation as inflexible in relation to fluctuating health and argued that RTW would be facilitated if each cancer survivor could determine their own scheduling. The regulation was considered complicated, and its lack of flexibility could cause cancer survivors to choose full-time sick leave instead of trying to balance work with sick leave.

*Today it’s possible to be on sick leave part-time and work part-time, but then every time the person gets worse, a new application for a change in sick leave is needed and that can...there’s so much administration around it, even the person has to keep track, so in the end you don’t do it*.

[14, Social Insurance Agency]

Informants noted that sick leave was generously granted during the first year, with minimal administrative demands to avoid burdening cancer survivors. Few questions were asked by the Swedish Social Insurance Agency during that period, and the prevailing attitude was that cancer survivors should not be bothered with routine bureaucracy, which could add unnecessary stress during an already challenging time. There were also certain circumstances that allowed cancer survivors to be on sick leave for special reasons. Furthermore, if the treatment is ongoing, this patient group does not need to be assessed for all work in Sweden.

*As long as cancer treatment is ongoing, the person doesn’t have to be assessed in relation to all jobs in Sweden. So that exception we have is great*.

[16, Social Insurance Agency]

The advantages and disadvantages of the ease of getting sick leave during the first year were discussed as something that could prolong sick leave. However, they believed that the real problem comes after patients have been on sick leave for 550 days and are fully covered by social insurance. At this point, cancer survivors need to be matched against the entire labor market, which can be a problem for many.

### Views and Expectations of the Individual Effect of RTW

In the interviews, stakeholders on the labor market emphasized that, in several respects, individual conditions affect RTW. They said that this was a matter of providing support, having empathy, and calibrating when a push is needed to help the individual summon up the strength or courage to return to work. It is also about helping the individual feel included in the social interaction that a workplace can provide.

#### Courage to Push and Make Demands

There was a perception that individuals on sick leave sometimes needed encouragement to avoid getting stuck in their situation. If people have been on sick leave for a long time and have completed all treatment phases, they might need a gentle push out of their comfort zone to regain the confidence to return to work. This encouragement was seen as crucial in determining whether the individual would dare to take the step back into working life.

*And then the healthcare system plays a very important role and then maybe they need to be the ones who push patients a bit “but now you need to start thinking about your job.” Instead of prolonging and prolonging and prolonging the sick leave period.*

[16, Social Insurance Agency]

The informants reported finding it easy to fall into the role of feeling sorry for the person on sick leave. However, they did not believe that this was beneficial to people on sick leave, as they could easily adopt that role. Therefore, they emphasized the importance of not allowing the person on sick leave to remain in that condition for too long, as it could lead to passivity. It was mentioned that laying out a clear plan for RTW, with realistic requirements, was beneficial. The informants believed that applying pressure was necessary, although it should be well balanced and based on the patient’s situation and well-being.

*I think having support, understanding and a little push from people in a person’s life is helpful. But it should take into account the treatment stage and how difficult the treatment is*.

[12, Social Insurance Agency]

The informants also emphasized the need to be less afraid of providing that extra push. This approach, they noted, should be carried out with care and consideration for the patient’s individual conditions, while still daring to challenge and motivate the patient when appropriate.

#### Inclusion in the Workplace

The informants discussed the importance of including cancer survivors in the workplace as part of health promotion work. They described various approaches to achieving this. Some informants described a long and balanced process to maintain social contact with the person on sick leave, such as regular monthly check-ins via SMS text messages to later invite them to lunch or breakfast. Over time, they might encourage the individual to visit the workplace and participate in social events, for example, by attending a shared 30-minute coffee break.

*We met regularly. At first maybe every three months or so // --- // And then it was more like: How are things going? How do you feel? When do we think it’s time to start working?*

[22, manager]

The informants emphasized the importance of people on sick leave maintaining contact with their work group and participating in social activities. Otherwise, they noted, it could feel like walls were being built, making it difficult to reconnect after a long absence. Managers also described how they would check in with the person on sick leave beforehand to understand their preferences regarding communication about their cancer diagnosis. They had experience not only with coworkers who wanted to talk openly about their diagnosis but also with those who found it too difficult to discuss in social settings. By gathering this information, managers could help prepare other employees for interacting in a way that aligned with the sick person’s wishes. They highlighted the need for continuous adaptation, as preferences varied across individuals.

*I’ve sometimes just said “but can’t you just go back right after treatment and bring some cake and sit in the breakroom for 30 minutes just to get it over with?” so your first day back at work doesn’t mean working and taking care of everything. // --- // Most people who keep in touch throughout the whole period find it easier to return*.

[7, physician]

The informants also reported that challenging situations could arise, for example, when a replacement had to be brought in for the person on sick leave. This could make the person on sick leave feel threatened or uncomfortable. The informants stressed the great importance of not forgetting the person on sick leave but instead ensuring that they felt included in what was happening at work, even if they were physically unable to be there. They believed that this was a big step toward making the road back to work short when that time came.

### Emotional Significance of Work

In several interviews, stakeholders on the labor market described how individuals’ experiences and attitudes toward returning to work are, in many respects, based on emotions. This included the feeling of wanting to work and the social interaction that work can provide. Several concerns were also raised, such as the fear of working or not having the strength to do so.

#### The Desire to Work

There was a general opinion among informants that cancer survivors had a strong desire to work. In many cases, this was described as a deep-seated and significant aspiration. The informants reasoned that work was not only a source of income, but it also met important social and psychological needs. Being able to continue working creates a sense of belonging, strengthens identity, and promotes an experience of belonging to a group and being part of the healthy world.

*Most people want to work. I must say that’s a really exciting lesson I learned from working with people, that everyone is so eager to get back to work. In my experience*.

[2, occupational therapist]

The informants reported that most people who wanted to continue working or return to work after a cancer diagnosis often succeeded, even if the process was challenging. They said that it could be particularly difficult for those who had more physically demanding work compared with those who had lighter physical jobs. Moreover, they indicated that the status of the profession was important for the possibility of returning to work, although this aspect was not clear-cut.

#### Existential Questions Come Into Focus

The informants discussed existential questions as factors that could both promote and hinder RTW. The period following treatment was described as challenging for many patients with cancer. In addition to the physical challenges that could arise, existential dimensions could influence an individual’s ability to return to work. These dimensions included questioning the very purpose of work, feeling that work was meaningless, or that the job lacked purpose when you were facing uncertainty about your survival.

*Well, if you get a disease, cancer or something else resulting in sick leave, then the person gets caught up in that process, mostly alone, accepts the disease, but is maybe afraid of not surviving. That’s a limiting aspect*.

[20, manager]

In addition to existential concerns, the informants discussed the fact that cancer survivors might fear being infected by colleagues. During treatment, cancer survivors may have a weakened immune system, making the fear of infection very real, as an infection could jeopardize their ability to continue treatment. To address this, stakeholders reported adjusting work arrangements to minimize contacts with others, including enabling remote work. Such measures reduce commuting, lower infection risk, and allow survivors to manage their workload according to their capacity.

## Discussion

### Principal Findings

This study underscores that the RTW process for cancer survivors is a complex and multifaceted challenge, requiring coordinated efforts across the health care, insurance, and employment sectors. The interview findings reveal several key factors that act as either facilitators or barriers to successful RTW. Notably, the significance of interdisciplinary collaboration, clearly defined roles and responsibilities, and flexible support structures emerged as critical areas with considerable potential for improvement. However, these areas also pose challenges due to the different perspectives and priorities among stakeholders.

Building on this overall complexity, the findings underscore the need for enhanced collaboration and clearer role definition within the RTW process for cancer survivors, and in the planning of future RTW interventions. Despite a shared consensus among stakeholders to support individuals in their transition back to work, systemic barriers such as siloed organizational structures, ambiguous responsibilities, and insufficient communication were perceived to impede effective coordination. Similar challenges have been noted in other contexts. Svärd et al [38] observed that stakeholders in RTW processes for individuals with common mental disorders often operate under competing goals and ambiguous mandates, leading to fragmented efforts. In this study, stakeholders expressed expectations of other actors while remaining uncertain about their own responsibilities. This finding is noteworthy given Sweden’s rehabilitation coordinator role, designed to support individuals on sick leave and facilitate coordination among stakeholders, including health care professionals, employers, and social insurance officers. Strengthening the mandate and visibility of these coordinators may therefore improve coordination outcomes, and comparable roles could be valuable in other national contexts. Désiron et al [39] likewise emphasized that such coordinators can provide tailored support, maintain continuity, and help bridge gaps between stakeholders.

The reported lack of coordination and cooperation between stakeholders may be linked to how rehabilitation coordinators have been implemented in Sweden. Although such functions have been introduced and are present in many settings, previous research has indicated that their role is not yet consistently established or fully integrated into RTW processes [32,33]. Barriers such as unclear mandates, variable recognition among stakeholders, and limited resources may restrict their ability to fulfill their intended coordinating function [32,33]. This may partly explain why participants in this study continued to describe persistent gaps in coordination.

More broadly, previous research has emphasized the importance of facilitators in changing processes [1]. Strengthening collaboration and clarifying the division of responsibilities among stakeholders are essential for ensuring a smoother RTW experience. For instance, Lau et al [36] highlight that siloed health care systems impede information exchange and collaborative evidence generation, ultimately undermining patient outcomes and system efficiency. The lack of integration among health care providers, social insurance agencies, and employers results in duplicated efforts, delayed interventions, and increased frustration among professionals. In our study, participants consistently emphasized the importance of teamwork and interprofessional collaboration. This statement aligns with findings from Désiron et al [39], who advocated for a dedicated RTW coordinator to improve work-oriented rehabilitation interventions. In a previous interview study by Bilodeau et al [37], employer representatives’ perspectives on supporting breast cancer survivors in their RTW process were explored. These findings closely align with stakeholders’ perceptions in our study, reinforcing the notion that collaboration challenges, unclear responsibilities, and communication barriers are prevalent across contexts. The highlighted issues of stakeholder collaboration reflect our observations regarding fragmented efforts and unclear mandates.

In addition to these coordination challenges, stakeholders perceived that structural barriers, such as financial challenges and sick leave regulations, must be addressed when promoting cancer survivors’ RTW. Financial constraints often discouraged employers from accommodating employees who were not fully productive, reflecting the findings of Fitch and Nicoll [47], who identified reduced income as a significant adverse consequence for cancer survivors. Financial difficulties are often associated with increased psychological distress and reduced quality of life [48].

Employers frequently struggled with balancing economic considerations by providing suitable workplace adjustments, resulting in conflicts, redeployment, or even unemployment for cancer survivors. The complexity and inflexibility of sick leave regulations were also identified as critical issues. Cancer survivors faced significant administrative burdens when adjusting their sick leave arrangements, particularly regarding rules that mandated even distribution of working hours across the week. Such rigid regulations often discouraged survivors from attempting gradual RTW, potentially prolonging full-time sick leave unnecessarily. To address these barriers, simplifying sick leave regulations and providing clear, adaptable guidelines are essential. Additionally, introducing financial incentives for employers who facilitate flexible and partial RTW arrangements could encourage greater inclusivity and reduce economic pressures. These measures could significantly enhance the feasibility of accommodating cancer survivors in workplaces, promoting healthier, sustainable RTW.

Our findings underscore the importance of tailoring work conditions to cancer survivors’ individual needs during RTW. Key supportive factors were flexible, personalized arrangements; gradual workload increases; remote work options; and adaptable schedules. Limited, nonpressured testing of work capacity was particularly valued for balancing health and rebuilding confidence. Stakeholders across professions emphasized individualized support, reflecting the complexity of recovery. This aligns with López-Faneca et al [35], who highlight the value of personalized RTW programs, and Bilodeau et al [37], who emphasize humanistic management and flexibility. They also stress the importance of addressing unclear responsibilities and expectation gaps, as these may hinder coordination. Together, these findings underscore the need for structured measures such as clear communication, emotional support, respect for privacy, and tailored interventions. Overall, our findings reinforce the need for individualized, flexible, and coordinated RTW approaches to promote recovery, sustainable employment, and workplace inclusion.

Beyond workplace adaptations, stakeholders considered cancer type, health status, treatment duration, and family circumstances as influential for cancer survivors’ motivation and ability to return to work. Personal attitudes, expectations, and the emotional meaning of work were also emphasized. A workplace where individuals feel included and not defined by illness was viewed as essential, and encouraging workplace contact and inclusion was seen as an important aspect of stakeholder support, aligning with reviews indicating broad consensus that RTW should be encouraged when health permits [49]. Stakeholders further highlighted health-promoting factors, such as social belonging and adjusted workload, as important for sustainable RTW. This is in line with a previous review evaluating the importance of personal and social factors to sustainable RTW after ill health due to mental disorders or musculoskeletal disorders [34]. Furthermore, stakeholders stressed the need to gently give cancer survivors a push back to work, always with the cancer survivors’ well-being in mind. One study showed that strengthening people’s self-efficacy regarding RTW is an important factor for successful and more rapid RTW [50]. Earlier research has also shed light on the perceived importance of maintaining contact with work during sickness absence [51]. However, uncertainty about when to initiate communicating with employees and the fear of exacerbating cancer survivors’ condition by making contact were described by stakeholders in earlier research and perceived as barriers to RTW [51].

Extending these findings, the results showed that stakeholders clearly perceived that RTW can promote multiple dimensions of well-being for cancer survivors on sick leave. Social benefits include reestablishing connections with colleagues, a sense of belonging, and receiving collective support, while economic benefits involve transitioning from sick leave benefits to earned income. This multifaceted value of RTW is an important finding in this study. However, stakeholders may need to consider that RTW can serve as a coping mechanism for managing existential reflections associated with a potentially life-threatening illness. The transition back to work can help survivors regain a sense of normalcy and control [52], yet it may also mask underlying emotional distress [53]. Research shows that cancer survivors often experience heightened anxiety and depression after treatment ends, partly due to reduced contact with health care providers and lingering fears of recurrence [13]. These psychological challenges can be exacerbated in cases where recovery is delayed or complicated, increasing the risk of emotional setbacks. Therefore, RTW interventions must address both physical and emotional dimensions of recovery [35] and involve stakeholders not only from the health care and the Social Insurance Agency but also from employers, who are generally not involved in developing RTW interventions [54].

Taken together, these findings are in line with previous research on RTW across a range of chronic conditions [7,25,34], which consistently highlights the importance of coordination, communication, and role clarity among stakeholders involved in the RTW process [20,38]. Similar barriers, such as fragmented responsibilities, limited collaboration between organizations, and challenges in navigating complex systems, have been described in studies of other chronic disorders [36,50,54], suggesting that many of the identified themes reflect more general structural and organizational challenges in RTW processes.

At the same time, some aspects appear to be more specific to cancer. These include the unpredictability of the disease trajectory [9,53], the impact of treatment-related side effects such as fatigue and cognitive impairments [13,20,52], and the need for flexible and individually adapted RTW processes over time [10,21]. Such factors may place additional demands on both coordination and workplace adaptations and highlight the importance of addressing cancer-related needs within RTW interventions. Taken together, these findings suggest that while several barriers and facilitators are shared across diagnostic groups, effective RTW support for cancer survivors also requires attention to condition-specific challenges [22,34].

The findings also align with the model by Feuerstein et al [20], which emphasizes the need to tailor rehabilitation to the individual cognitive and emotional resources of cancer survivors and to the contextual demands they face. Although the analysis was inductive and not theoretically driven, the results underscore the importance of coordinated, individualized support across multiple systems. The complexity of the RTW process—requiring interdisciplinary collaboration, clearly defined roles, and flexible support structures—reflects the view of Feuerstein et al [20] that effective rehabilitation must integrate both personal and structural factors. Framed within this model, the study advances understanding of how rehabilitation practices and policies can better support survivors’ transition back to work.

Finally, the findings of this study have also informed the development of a planned intervention. In particular, the identified challenges related to coordination and communication suggest a need to strengthen structured collaboration between stakeholders. The importance of role clarity highlights the need to define responsibilities more explicitly within the intervention. Furthermore, the identified need for individualized and flexible support indicates that the intervention should allow for adaptation to individual circumstances and changing needs over time. Finally, the findings underline the importance of addressing both workplace-related and health care–related aspects within a coordinated framework.

### Strengths and Limitations

The study has several strengths, including the methodological approach, which was considered appropriate for understanding and describing perceptions. In addition, a purposive sampling strategy and multiregional representation ensured broad stakeholder perspectives and increased contextual diversity and potential transferability. The sample size was considered sufficient, reflected in the rich interview data. One explanation could be the use of a fictional case during semistructured interviews. This may have helped the participants reflect on realistic scenarios, while the semistructured format allowed for depth in responses. One limitation of the study is that some stakeholder groups were underrepresented, which may have limited the diversity and comprehensiveness of perspectives.

The sample comprised fewer participants from the Employment Service Agency compared with other stakeholder groups, which may limit the depth of insights from this sector. However, this may reflect the limited involvement of this stakeholder group in RTW processes for cancer survivors within the Swedish system. Nevertheless, the sampling strategy was designed to maximize variation across key roles rather than to ensure equal group sizes, and we gathered substantial input from representatives in health care, social insurance, and employer organizations. Moreover, stakeholders were asked about cancer survivors as if they were a homogeneous group. This may be a limitation, because although rehabilitation and the conditions for RTW after cancer vary depending on the cancer diagnosis and severity, the tasks to be performed at work also vary.

A further limitation of the study is that the study did not specifically investigate stakeholders’ experiences of rehabilitation coordinators or their expectations regarding this function. Given the ongoing implementation of coordination efforts, future research should examine how rehabilitation coordination is perceived and how it can be further developed to support RTW processes.

In addition, cancer survivors were not included as participants. As key actors in RTW processes, their perspectives are essential for a comprehensive understanding. However, the study was designed to focus on stakeholders in working life, and the findings should be interpreted in light of this scope. Previous studies by our research group have explored cancer survivors’ perspectives, which complement the present findings.

### Implications and Further Research

Building on these findings, a clearer definition of roles and shared responsibilities among working-life stakeholders is essential to effectively support the RTW process among cancer survivors. Flexibility within the RTW process is crucial for enabling individuals to resume work at their own pace, facilitating their transition out of illness-related isolation, and promoting social interaction with colleagues. These are important aspects to consider in future policy development, interventions, and support measures. Additional research is needed to explore cancer survivors’ personal RTW experiences, with the goal of obtaining a more comprehensive understanding of the factors that influence the RTW process. To support interpretation and guide future research, Table 3 summarizes the implications of the findings for the Swedish RTW system.

**Table 3.** Summary of the implications of the findings for the return to work system.

Findings	Implications
A desire for collaboration and clear division of responsibilities	Ensure that each stakeholder has a clear understanding of their respective roles and responsibilities. Ensure clarity regarding the division of responsibilities. Appoint a dedicated person to oversee and support the entire RTW^a^ process.
Balancing individual adaptations	Gradually increase workload tailored to needs and work conditions. Flexible RTW plan, developed with the cancer survivor. Allow, if possible, adaptations to the work situation.
Reducing structural barriers through support	Stakeholders emphasized the need for a structured collaboration plan to clarify roles and enhance teamwork. Promote awareness about available support. Check progress along the way.

a RTW: return to work.

### Conclusions

This study highlights strategies that facilitate a successful RTW process among cancer survivors from the perspectives of working-life stakeholders. The findings underline the importance of individualized and tailored support, as well as the need for clear policies to guide stakeholders’ roles and responsibilities. Clarifying the rehabilitation coordinator’s role in improving stakeholder cooperation, and enhancing knowledge of cancer survivors’ specific workplace needs, can collectively facilitate a smoother and more sustainable RTW transition.

## Supplementary material

10.2196/89954 Multimedia Appendix 1 Case description used in the interviews to guide discussions on return to work, translated from Swedish.

10.2196/89954 Checklist 1 COREQ (Consolidated Criteria for Reporting Qualitative Research) checklist.

## Abbreviation

AI: Artificial Intelligence
COREQ: Consolidated Criteria for Reporting Qualitative Research
RTW: return to work
