# Supplementary material for: Stakeholder Perspectives on Cancer Survivors’ Return to Work and Well-Being: Qualitative Interview Study
Source: JMIR Cancer. 2026 May 11;12:e89954. doi: 10.2196/89954 (PMC13160487; doi:10.2196/89954)
Supplement: Multimedia Appendix 1 [file cancer-v12-e89954-s001.docx]

**Multimedia Appendix 1.** Case description used in the interviews (translated from Swedish).

Kim is currently undergoing cancer treatment and is of working age.


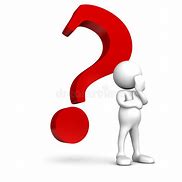


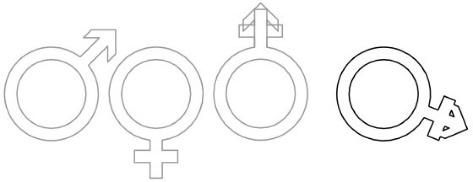


The person in this case may experience challenges related to both work and health during and after cancer treatment. This could include difficulties staying at work during treatment, returning to work afterward, maintaining employment, finding a new job, or being financially self-sufficient. We would like you to reflect freely based on your role.
